# Improving hexaminolevulinate enabled cancer cell detection in liquid biopsy immunosensors

**DOI:** 10.1038/s41598-021-86649-6

**Published:** 2021-03-31

**Authors:** Kit Man Chan, Jonathan Gleadle, Jordan Li, Thomas Danny Michl, Krasimir Vasilev, Melanie MacGregor

**Affiliations:** 1 Department of Engineering, UniSA STEM, University of South Australia, Mawson Lakes, SA 5095 Australia; 2 Department of Renal Medicine, Flinders Medical Centre, Bedford Park, SA 5042 Australia; 3 College of Medicine and Public Health, Flinders University, Bedford Park, SA 5042 Australia; 4 Future Industries Institute, UniSA STEM, University of South Australia, Mawson Lakes, SA 5095 Australia

**Keywords:** Bladder, Microfluidics, Cancer screening, Urological cancer, Biosensors, Biological techniques, Cancer, Biomarkers

## Abstract

Hexaminolevulinate (HAL) induced Protoporphyrin IX (PpIX) fluorescence is commonly used to differentiate cancer cells from normal cells in vivo, as for instance in blue light cystoscopy for bladder cancer diagnosis. A detailed approach is here provided to use this diagnostic principle ex vivo in an immunosensor device, towards enabling non-invasive cancer diagnostic from body fluids, such as urine. Several factors susceptible to affect the applicability of HAL-assisted diagnosis in body fluids were tested. These included the cell viability and its impact on PpIX fluorescence, the storage condition and shelf life of HAL premix reagent, light exposure (360–450 nm wavelengths) and its corresponding effect on both intensity and bleaching of the PpIX fluorescence as a function of the microscopy imaging conditions. There was no significant decrease in the viability of bladder cancer cells after 6 h at 4 °C (student’s t-test: *p* > 0.05). The cellular PpIX fluorescence decreased in a time-dependent manner when cancer cells were kept at 4 °C for extended period of time, though this didn’t significantly reduce the fluorescence intensity contrast between cancer and non-cancer cells kept in the same condition for 6 h. HAL premix reagent kept in long term storage at 4 °C induced stronger PpIX fluorescence than reagent kept in the − 20 °C freezer. The PpIX fluorescence was negatively affected by repeated light exposure but increased with illumination intensity and exposure time. Though this applied to both healthy and cancer cell lines, and therefore did not statistically improved the differentiation between cell types. This study revealed important experimental settings that need to be carefully considered to benefit from the analytical potential of HAL induced fluorescence when used in technologies for the diagnosis of cancer from body fluids.

## Introduction

Cancer diagnosis and risk stratification tests are routinely performed using biopsy or cytology material obtained from primary tumors^1,2^. These methods present limitations that could be overcome by implementing liquid biopsy from body fluids instead. Indeed, biopsy has the inconvenience of being invasive which limits its use as follow-up tests for the response to treatment and disease progression^3^. On the other hand, cytopathology, relies heavily on detailed sample preparation, immunochemistry staining, and expert image interpretation^4^. These aspects largely limit cytology from reaching a definitive diagnosis^5^. By contrast, liquid biopsy is an emerging non-invasive diagnostic tool for the management of cancer patients which can provide information on the tumor-derived genomic profile^6,7^. It consist of detecting free cancer cells, cell-free DNA fragment or other biomarkers released from the tumor that are present in biofluid samples collected from the patient^8^. Compared to tissue biopsy, liquid biopsy has the advantage of being minimally invasive and can therefore be performed regularly to monitor disease progression and adjust treatment as necessary^8,9^. Whilst blood is the most common fluid used in the development of liquid biopsy approaches^10^, the method is versatile as any biofluid that contains biomarkers shed by the tumor can be used. These include cerebrospinal fluid, saliva, urine and other bodily fluids^10,11^. Another versatile aspect of liquid biopsy is the varying type of biomarkers it targets, which include but are not limited to circulating cell-free tumor DNAs (ctDNA) and RNAs (ctRNA), cell-free proteins, exosomes, messenger RNA (mRNA) as well as whole circulating tumor cells (CTCs). The latter can reveal the tumor prognosis and heterogeneity, and is therefore a target of choice in the development of point of care devices for early detection of metastasis and personalized treatment^12–14^. However, in order to fully harvest the benefit of a liquid biopsy approach targeting CTC, several of the above-mentioned limitations of cytology need to be addressed, specifically easing the sample preparation step and automating the image analysis^15^. This challenge is here tackled using the specific case of bladder cancer detection.

In previous work, we developed a microfluidic device capable of capturing and identifying bladder cancer cells from the urine of patients^16–19^. Bladder cancer is the 10^th^ most common cancer and has one of the highest recurrence rates^20,21^. As a result, patients must undergo regular surveillance via cystoscopy every 3–6 months for two years, every 6–12 months for years 3–4, and at least annually after that. Cystoscopy is an invasive surgical procedure with many complications^22,23^. In contrast, a liquid biopsy of urine would allow for the frequent and pain free tracking of recovering bladder cancer patients. Bladder cancer is also a good model for the broader development of liquid biopsy methods because malignant cells are readily shed in urine, a non-invasive biofluid which is a far less complex fluid than blood containing much less satellite cells^24^.

The technology we developed for the specific detection of bladder cancer cells shed in urine combines microfluidic immunocapture with photodynamic diagnostic principles. Specifically, urine is passed through a plasma polymer coated microfluidic chamber immunofunctionalised with anti-EpCAM antibodies^17^. The immunofunctionalisation is capable of withstanding the harsh urine environment (high ionic strength, acidic) thanks to the plasma deposited polyoxazoline coating (POx) used to covalently bind bioactive anti-EpCAM antibodies, as previously demonstrated^25,26^. The captured cancer cells are then distinguished from healthy cells by using Hexaminolevulinate (HAL) which induce cancer specific protoporphyrin IX fluorescence^27^. HAL is a derivative of 5-ALA, a precursor of protoporphyrin IX (PpIX) in the heme biosynthetic pathway^28^. The exogenous administration of 5-ALA increases the endogenous accumulation of PpIX preferentially in tumor cells of various origin^19,29,30^. Factors contributing to the preferential accumulation of PpIX in tumor cells include the altered activity of PpIX precursors and iron transporters, reduced ferrochelatase (FECH) expression, altered glucose metabolism and reduced nicotinamide adenine dinucleotide phosphate (NADPH) in tumour environment and oncogenic mutations^28^. Since PpIX is a fluorescent compound, the administration of 5-ALA and its derivative has been used in photodynamic cancer diagnosis (PDD) and therapies (PDT). For bladder cancer, HAL-induced PpIX fluorescence is the cornerstone of blue light cystoscopy. Compared to white light cystoscopy, it enables better visualization of the tumors for biopsy, diagnosis, and more complete surgical resection of bladder lesions^31^. Our technology aims to transfer the principle of PDD to the single cell level in a non-invasive, liquid biopsy type of cancer diagnosis, which could ultimately be used for different type of malignancies detected in various body fluids, as suggested by our preliminary works with prostate cancer^19,26^.

**Figure 1:**
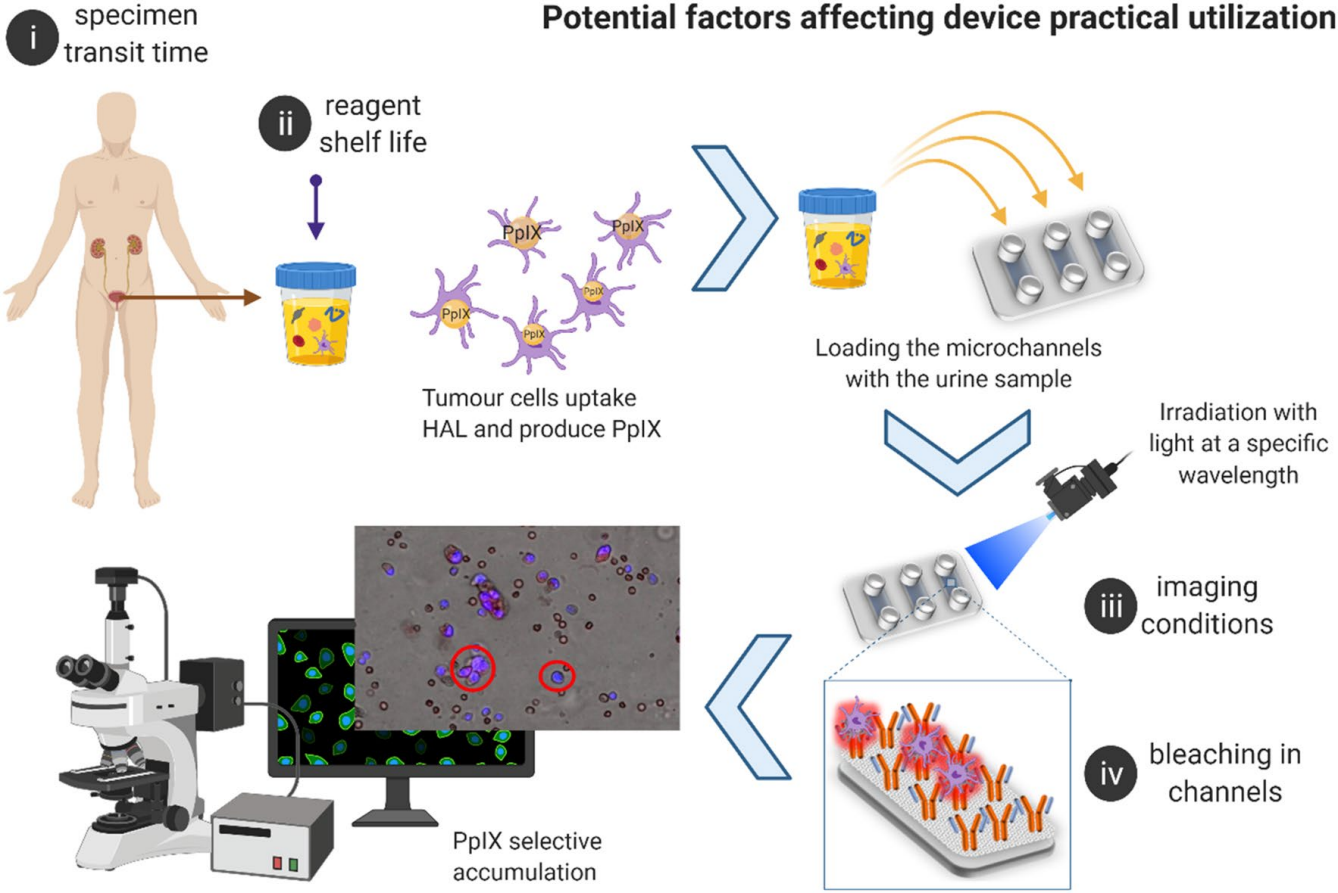
Infographics of the proposed diagnostic device and potential factors (**i**–**iv**) affecting practical utilization. Created with BioRender.com.

While the technology achieved 96% specificity and 100% sensitivity in a spiked urine proof of concept experiment^17^, hurdles arose when patient samples were examined^32^. Indeed, the underlying mechanisms responsible for the specificity of PpIX accumulation in tumor cells remain not well understood. In particular, the history of the cells shed in the urine appeared to alter their capability to produce PpIX. Specifically, four aspects of the practical usage of this technology were identified as being critical in the functioning of the device as schematized in Fig. 1i. The influence of urine storage conditions on cell viability and corresponding HAL-induced PpIX fluorescence. Figure 1ii. The shelf life of the reagents. Figure 1iii. The imaging conditions, in particular exposure time and intensity to maximize the specific PpIX fluorescence. Figure 1iv. The effect of light exposure on the photobleaching of the PpIX fluorescence. In this work, each factor has been investigated and optimized using cultured cell lines as models.

In defining these optimum conditions, this work identifies recommended conditions of use for the original technology as it moves from cell lines experiments to clinical trials. The results obtained here for the specific case of bladder cancer could inform decisions associated with the development of other liquid biopsy protocol using HAL-induced fluorescence for the diagnosis of other cancers ex vivo^33–36^.

## Results

### Cell viability and effect on HAL-induced PpIX fluorescence

**Figure 2:**
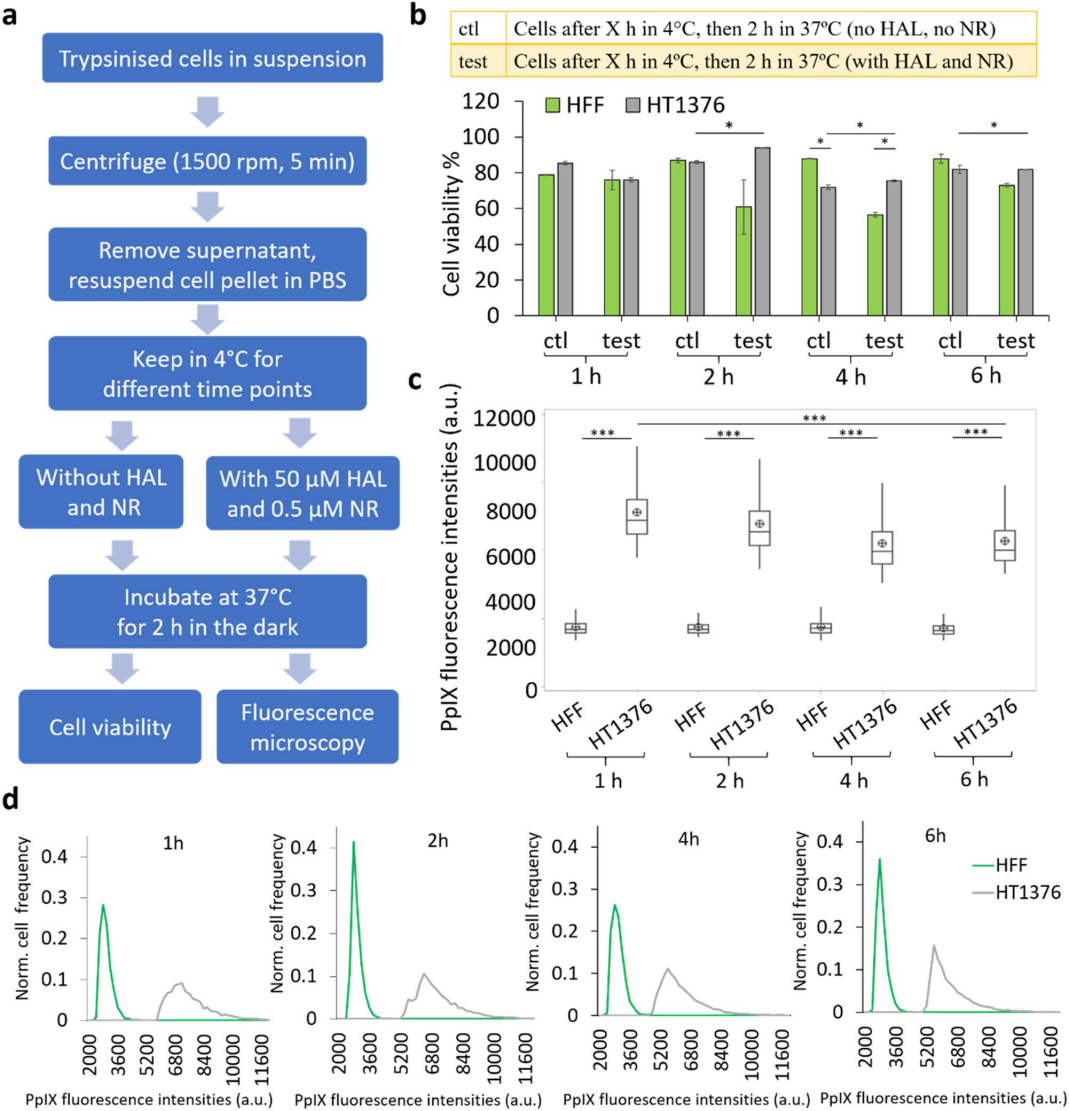
Cell viability and corresponding HAL-induced PpIX fluorescence for cells kept at 4 °C for 1 to 6 h before incubation with 50 µM HAL and 0.5 µM nuclear red. (**a**) Flow chart of the experimental protocol. (**b**) Cell viability result (*p < 0.05). (**c**) Boxplot illustrating the corresponding HAL-induced PpIX fluorescence for both cell lines, showing a statistically significant difference between normal fibroblast HFF and bladder cancer HT1376 cells (***p < 0.001) as well as between 1 and 6 h time points for HT1376 cells. (**d**) Histogram showing the normalized distribution of PpIX fluorescence intensity in normal fibroblast HFF (green) and bladder cancer HT1376 (grey) 4 °C after 1, 2, 4 and 6 h at 4 °C.

In a clinical setting, the sample collection would usually take place at the hospital and the specimen would typically be sent to a pathology laboratory for analysis. The time delay associated with specimen transportation may vary and have an impact on the sample quality and test results. These effects were evaluated here using cultured cells, in order to assess the feasibility of delayed analysis in practical situations. A potential way to use the technology for cancer cell detection is to add the HAL reagent to the urine specimen in the pathology lab, after it has been kept at 4 °C for transport. This scenario was assessed with cells lines left to incubate in PBS in controlled laboratory conditions. Cell viability was examined under two different conditions: (i) control (ctl): cells kept in 4 °C PBS for 1, 2, 4 and 6 h then heated back to 37 °C for 2 h (without HAL and nuclear red); (ii) test: cells kept in 4 °C PBS for the same amount of time then incubated with HAL and nuclear red at 37 °C for 2 h (Fig. 2a). From the 2 h’ time point, a reduction in cell viability was observed in the normal fibroblast HFF cells tested with HAL when compared with the control group. In contrast, there was no significant decrease in the viability of bladder cancer HT1376 cells for both the control and test groups, indicating that there was no cytotoxic effect for the bladder cancer HT1376 cells due to HAL at any of the time points investigated (Fig. 2b).

In contrast, following the different incubation times at 4 °C, the bladder cancer cells HT1376 displayed greater variation in PpIX fluorescence intensity than the normal HFF cells. The HFF had consistently low levels of PpIX fluorescence for all conditions while the PpIX fluorescence intensity of HT1376 decreased when the cells were kept at 4 °C for increasing periods of time (Fig. 2c). Specifically, the fluorescence of HT1376 is significantly lower after 6 h spent at 4 °C than after 1 h. Nonetheless, the PpIX fluorescence produced by HT1376 remained significantly higher (*p* > 0.001) than HFF for every single time points investigated. The shape of the histogram profile provides additional information on the PpIX fluorescence in each condition of both cell types. A distinctive separation was observed between the fluorescence intensity distribution of normal HFF and that of cancer HT1376 cells. A sharp peak occurs at an intensity value of about 3000 a.u for the normal HFF. For cancer HT1376 cells, the peak broadened out with a maximum shifted towards higher fluorescence intensities than the HFF peak. The histogram shows that there are two separated peaks at different fluorescence intensities. It is apparent that it is possible to discriminate between normal and cancer cells even when cells have been kept at 4 °C for 6 h (Fig. 2d). Nonetheless, shorter time at 4 °C gives a better separation and higher absolute fluorescence intensity for cancer HT1376 cells. It is worth noting, that storage at higher temperatures was not tested here because it has been shown to decrease the cell immunocapture efficiency^37^.

It was found that the PpIX fluorescence intensities measured in HT1376 decreased with time even though the cell viability did not. These results indicate that the relationship between cell viability and the level of PpIX fluorescence is not straightforward. Specifically, the lower fluorescence intensity observed at the 6 h time point could be due to the elution of PpIX out of the cell^38,39^, rather than a lack of cell metabolic activity. Overall, the data also shows that HT1376 can be distinguished from healthy HFF based on the HAL-induced fluorescence levels, even after 6 h incubation at 4 °C. These results from cell line experiment suggest that technologies using ex-vivo HAL-induced fluorescence could allow up to 6 h for transport of the cell suspension specimen without adverse effect on the detection sensitivity, providing that the cells remain metabolically active enough in the body fluid to produce PpIX.

**Figure 3:**
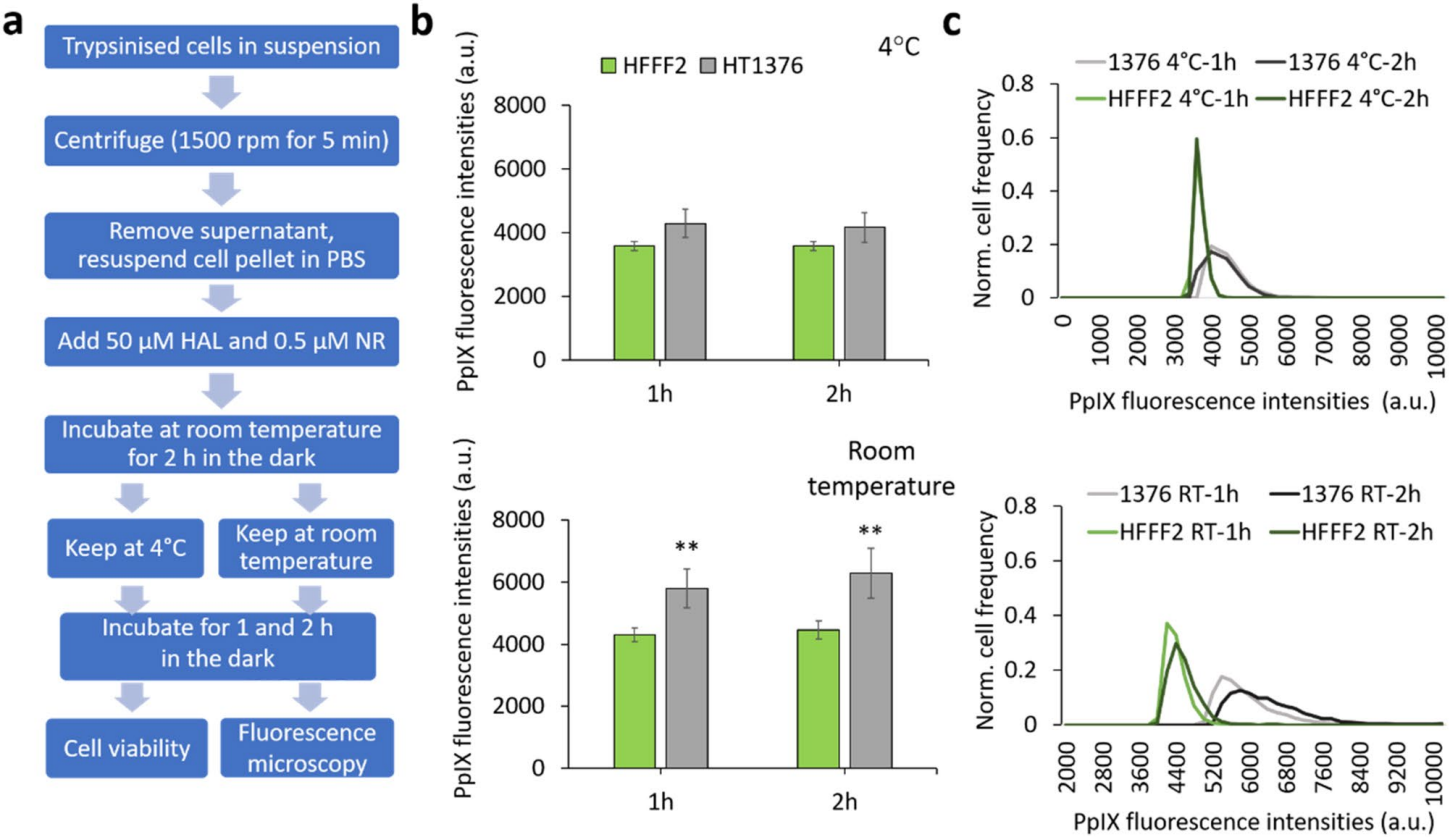
The effect of temperature on HAL-induced PpIX fluorescence when cells kept at 4 °C or room temperature after 2 h incubation of 50 µM HAL and 0.5 µM nuclear red. (**a**) Flow chart of the experimental protocol. (**b**) Bar chart illustrating the corresponding HAL-induced PpIX fluorescence for both cell lines, showing a statistically significant difference between normal fibroblast HFF and bladder cancer HT1376 cells (**p < 0.01) when cells were kept in room temperature (bottom) but not in 4 °C (top). (**c**) Histogram showing the normalized distribution of PpIX fluorescence intensity in normal fibroblast HFF and bladder cancer HT1376 for 1- and 2-h time points in 4 °C (top) and room temperature (bottom) respectively.

The second possible way to use the technology is to add the HAL reagent to the specimen at the time of collection, later cooling the specimen for transport to the pathology lab, therefore applying a temperature gradient to the specimen once the HAL has been added. This scenario was also tested and it showed that the temperature gradients had a negative effect on PpIX expression. In this test, the trypsinised cells were treated with mixed reagents for 2 h at room temperature first before cooling them down to 4 °C for 1 and 2 h, respectively. The results were compared to cells which remained at room temperature for the same amount of time. The results show that the fluorescence decreased by 35% and 51% for cells kept at 4 °C for 1 and 2 h, respectively, compared with cancer cells kept at room temperature (Fig. 3). Also, the difference in fluorescence intensity between cancer and HFF cells kept at room temperature for a full 4 h after adding the HAL reagent mix was not as significant as in the first scenario. This is attributed to the difference in the temperature at which the HAL incubation occurred. In the first scenario, the HAL incubation step takes place in a laboratory facility, where the specimen can be placed at 37 °C, while in the second scenario, the HAL incubation takes place at room temperature at the point of collection where such infrastructure may not be readily available. We therefore recommend that in a trial of clinical samples, the HAL reagent mixture be added in the pathology lab, after transport at 4 °C where necessary.

### Shelf-life of premix reagent for different storage temperature

**Figure 4:**
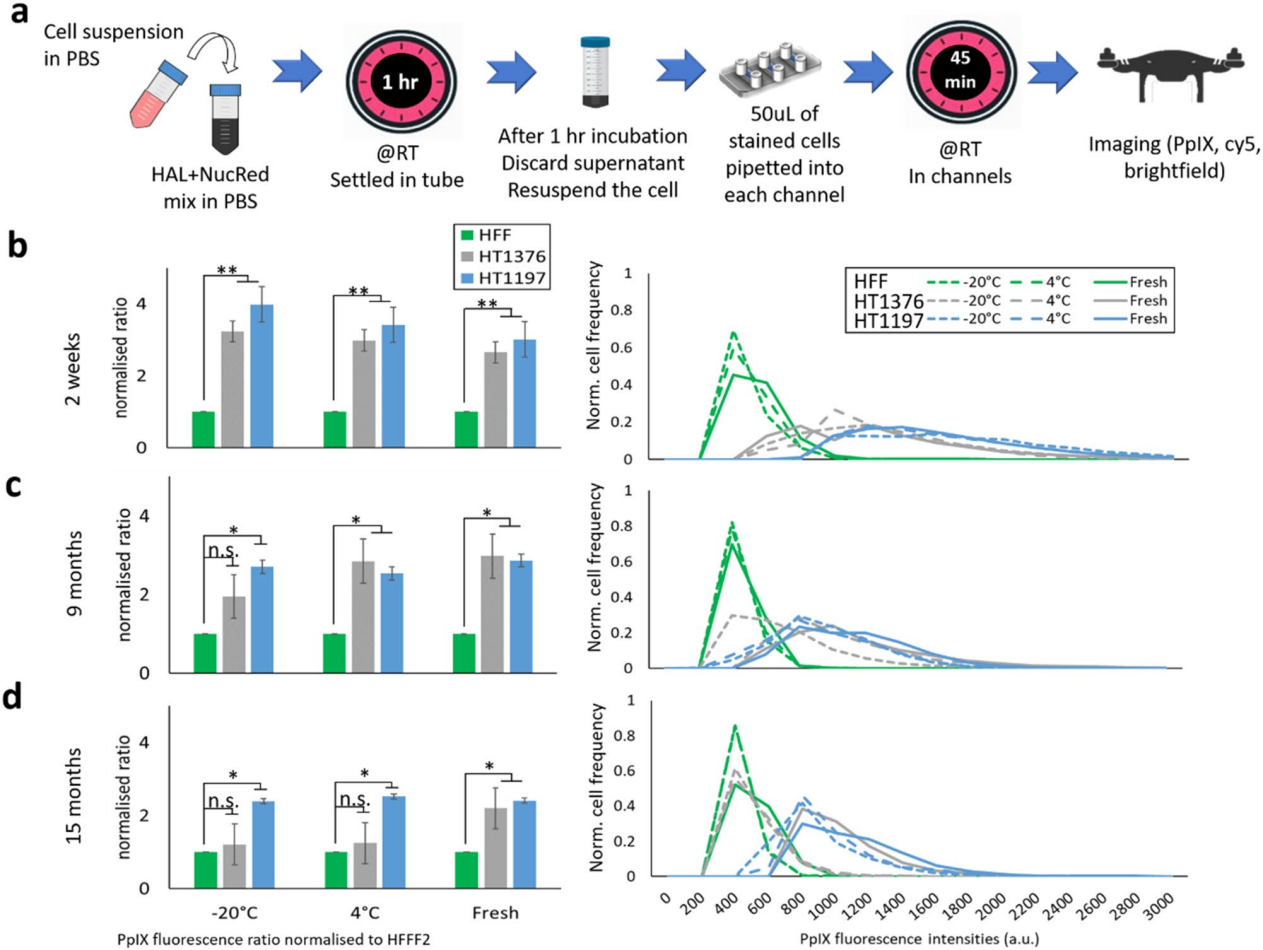
The effect of reagent mix storage temperature and storage duration (2 weeks, 9 months and 15 months). (**a**) Flow chart of the testing protocol. (**b–d**) Mean PpIX fluorescence ratio and normalised fluorescence histogram distribution for normal fibroblast HFF and two bladder cancer HT1376 and HT1197 cells treated with premixed HAL and nuclear red reagent stored at different temperatures for (**b**) 2 weeks, (**c**) 9 months and (**d**) 15. n.s. = not significant, *p < 0.05, **p < 0.01.

The effects of storage temperature and time on the premix reagent was determined and are shown in Fig. 4. The reagent mix consists of 50 µM of HAL and 0.5 µM of nuclear red in 1X PBS. It was stored either in a freezer at − 20 °C or in a refrigerator at 4 °C for up to 15 months. A reagent mix solution was freshly prepared from the commercial HAL (supplied in powder state) on the experiment day to be used as control.

For premix reagents stored for 2 weeks, the PpIX fluorescence intensities measured with the fresh reagent mix were comparable to those measured for reagent stored at both − 20 °C and 4 °C. The difference of PpIX fluorescence ratio between both cancer cell lines and HFF cells was statistically significant (*p* < 0.01) in all cases. However, after 9 months storage, using the reagent mix kept at − 20 °C led to a reduction in the PpIX fluorescence of HT1376. In which case the difference between normal HFF and HT1376 was no longer significant. In contrast, using reagents kept at 4 °C for 9 months, it was still possible to distinguish both cancer cell line from the control HFF. After 15 months, lower levels of PpIX fluorescence were measured for both HT1197 and HT1376 when treated with either stored premixes compared to the fresh reagent mix. Once again, this effect was more pronounced for HT1376, where the difference with HFF was no longer significant, in good agreement with what was observed at the 9 months time point. This indicates that HT1376 cells are more sensitive to the effect of long-term storage on the premix reagents than HT1197.

According to the product information statement provided by suppliers, some chemicals, typically those sold without a specific expiry date, are stable for extended periods of times. HAL, for instance, is sold in a powder form without an expiry date. However, for the diagnostic device assay to be compatible with point of care testing, the reagents would ideally be provided as a premix liquid, in a ready-to-use form suitable for use by unexperienced operators. This study shows that when HAL is reconstituted in PBS its efficiency for photodynamic cancer cell detection is limited to 9 months depending on the cell type investigated. PBS is widely used as a buffer solution and is biocompatible. The onset of crystallization in frozen PBS have been previously reported to cause significant pH shifts^40,41^. However, there was, to the best of our knowledge, no pre-existing evidence showing that premix of this reagent had a negative effect on the cellular PpIX fluorescence signal overtime. All the premix reagents used in this study were prepared at the same time and from the same lots of chemicals. We observed that in HT1197, a steady PpIX fluorescence performance was established in both premixes and fresh mix. The observed result suggested that the differences were related to the cell types. Both HT1376 and HT1197 are human bladder transitional carcinoma cells. HT1376 is derived from stage ≥ pT2 and grade 3 tumor, while HT1197 is derived from stage pT2 and grade 4 tumor. Several cancer-associated genes—Fibroblast Growth Factor Receptor-3 (FGFR3), phosphatidylinositol-4,5-bisphosphate 3-kinase catalytic subunit alpha (PIK3CA) and N-ras proto-oncogene (NRAS) are identified as mutant in HT1197 when compared to HT1376^42^. The difference of tumor grade and gene expression could be the reasons why these particular cells types responded differently to the aging premixes. Further molecular studies investigating the effect of these genes on the heme biosynthesis pathway (e.g. porphyrin biosynthesis enzymes) are warranted in order to fully understand the breath and limitation to HAL-induced fluorescence as a tool to specifically distinguish different cancer cells types from the variety of healthy cells they are mixed with in urine.

### PpIX fluorescence in test microchannels and effect of double light irradiation

A major shortcoming of 5-ALA and its derivatives for PDD is the fast photobleaching of PpIX under light irradiation, as previously reported^43,44^. Yet, up to now, the device performance had been determined using two set of images^32^. The first set of images is taken immediately after introducing the specimen into the microchannel. Cells counts from this “before rinse” images provide a benchmark of the original cellularity of the samples. After the set incubation time, the microchannels are rinsed with PBS and a second “post rinse” set of images is taken. The difference in PpIX positive cells between these two set of images is used to calculate the capture sensitivity. However, considering PpIX sensitivity to photobleaching^45^, this method could in fact affect the overall technology capability to detect cancer cell in patient urine specimens.

Another important aspect highlighted in our previous works is that the PpIX fluorescence differed for cells in suspension compared to cells in monolayers when treated with HAL ex vivo. Specifically, the PpIX fluorescence measured in cancer cells in suspension was much higher than that of cancer cells in cultured monolayer under the same incubation conditions^18,46^. These results suggested that the nature of the cell-surface interaction influences the cellular PpIX fluorescence performance. By extension, one cannot assume that the PpIX fluorescence intensity would be comparable in the microchannel where the cells are bound via immunoaffinity, i.e. to the anti-EpCAM functionalised test channel, and the positive control POx microchannel where cell bound in a non-specific manner. To test these hypotheses, we evaluated the PpIX fluorescence of cells captured on the two different microchannel surfaces (POx and anti-EpCAM) with or without double light irradiation.

**Figure 5:**
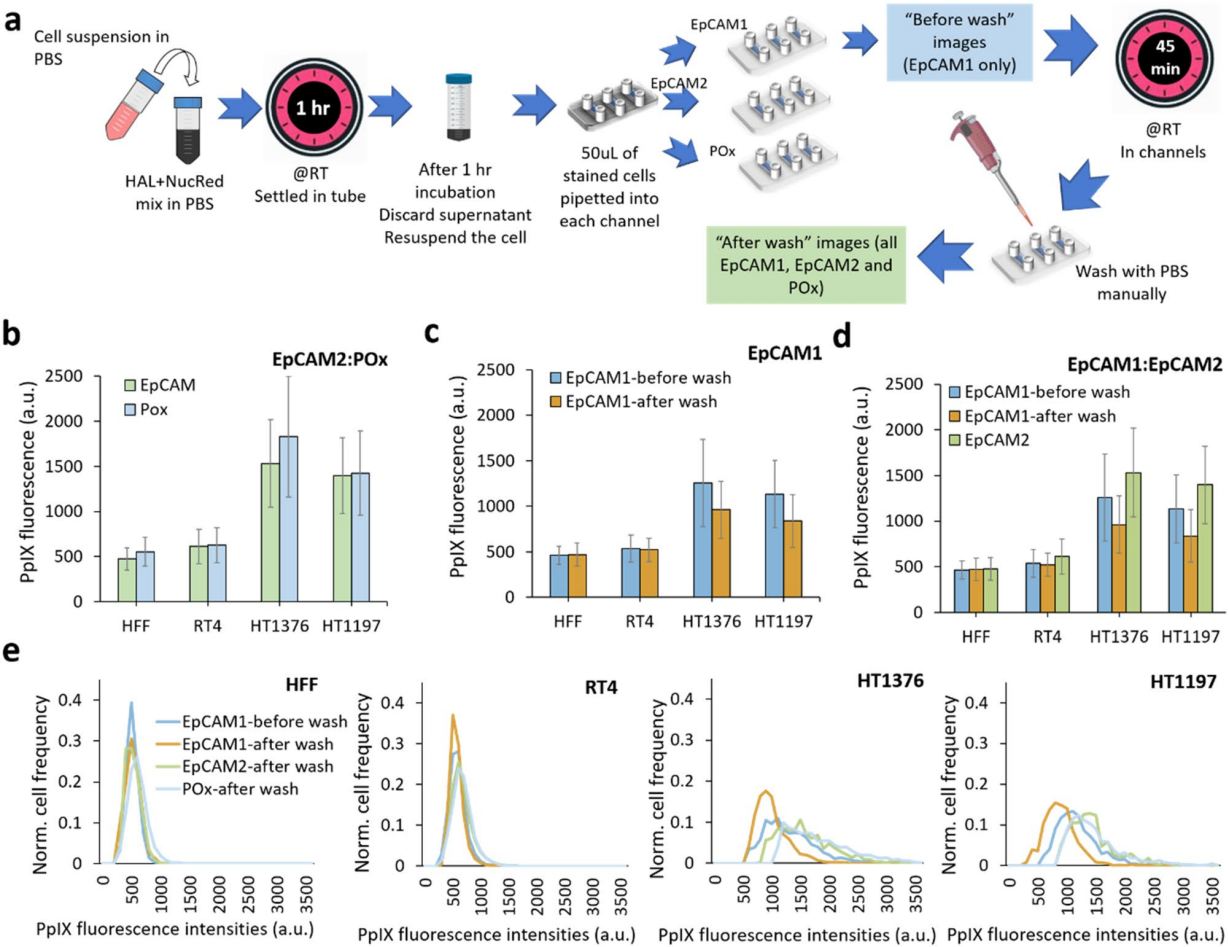
HAL-induced PpIX fluorescence of four different human cell lines in suspension on anti-EpCAM functionalised POx coated (EpCAM) and non functionalised POx coated (P) channels. (**a**) Experimental setup. The differences of PpIX fluorescence measured between (**b**) EpCAM2 and POx slide P; (**c**) before wash and after wash in EpCAM1, and (**d**) single blue light exposure in EpCAM2 and double blue light exposure in EpCAM1. (**e**) Normalised histogram profiles of all tested conditions.

A schematic of the experimental protocol is shown in Fig. 5a. Normal fibroblast HFF and three types of bladder cancer cells were examined under three conditions referred to as POx, EpCAM1 (exposed once) and EpCAM2 (exposed twice). First, we compared the fluorescence of cells adhered to the anti-EpCAM functionalised slide EpCAM1 to the fluorescence of cells adhered to the non-functionalised POx slide. In parallel, we compared single exposure to double exposure, by measuring the fluorescence intensity of cells in EpCAM1 and EpCAM2.

To compare HAL-induced PpIX fluorescence between EpCAM and POx channels, the EpCAM1 and POx slides were treated with the same procedure which only included a single blue light exposure. The number of cells bound on each channel before and after wash is used to determine the capture sensitivity of the device. The capture rate was 91% for RT4, 89% for HT1197, 87% for HT1376 and 1% for HFF in this spiking experiment. The results show that there is no significant difference in PpIX fluorescence intensity in any cancer cell lines between the EpCAM and POx channels (Fig. 5b). We can therefore conclude that the different adhesion mechanism at play in POx (nonspecific adhesion) and antibody functionalized channel (immunoaffinity) do not affect the HAL-induced fluorescence levels measured after washing off loosely bound cells.

For the purpose of assessing the impact of double blue light exposure on the intensity of PpIX fluorescence in cells (Fig. 5b–d), cells in the EpCAM2 channels were imaged before and after rinse. For cells expressed low PpIX fluorescence such as non-cancer HFF and bladder cancer RT4, double blue light exposure had no effect on their PpIX fluorescence. Although the differences were not significant for any of the bladder cancer cell lines investigated, a decrease in fluorescence intensities caused by photobleaching was observed in both HT1376 and HT1197 cells. Next, we compared the double (EpCAM2) and single (EPCAM1) exposure of blue light illumination conditions. As expected, cells in EpCAM1 slides which were only imaged once after rinse, show higher PpIX fluorescence than those in EpCAM2. (Fig. 5e). Hence, we recommend that in future trial, patient specimen is only imaged after wash in order to obtain the maximum fluorescence measurement.

### PpIXfluorescence imaging optimization

**Figure 6:**
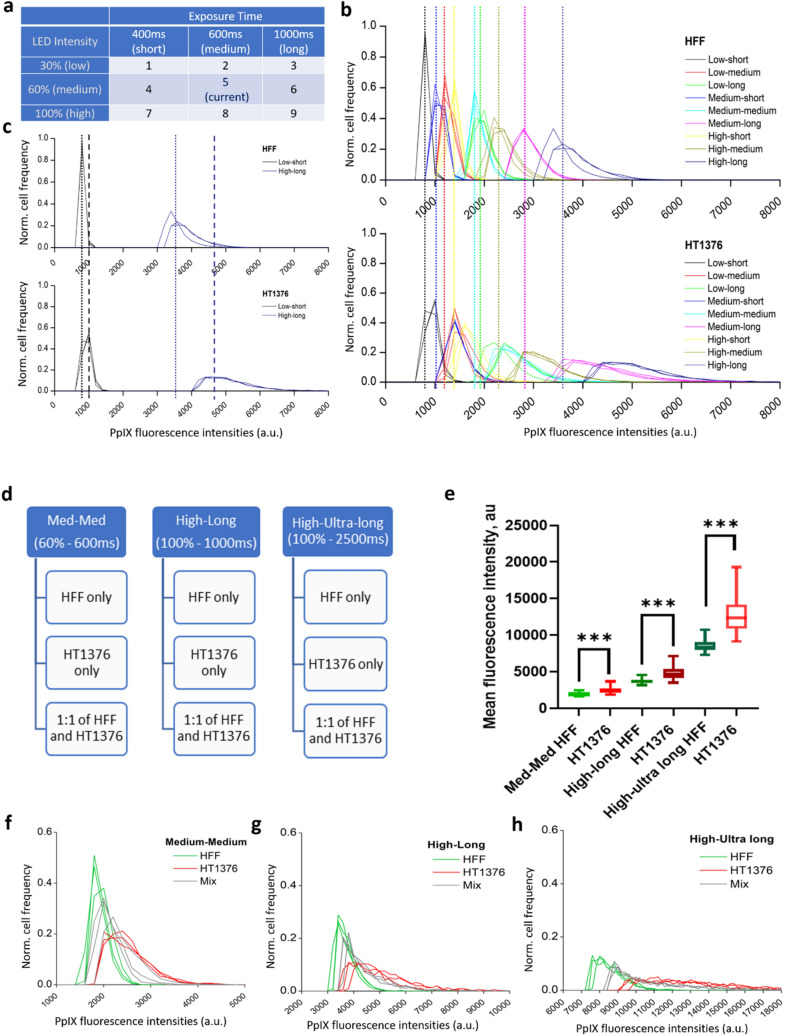
The effect of exposure times and LED illumination intensities on PPIX fluorescence. (**a–c**) for the primary optimization stage. (**a**) systematic configuration for PpIX luminescence imaging, nomenclature: illumination intensity—exposure time (e.g. low-long); (**b**) the representative PpIX luminescence spectrum of HFF (top) and HT1376 (bottom) in all configurations; (**c**) comparison between the most extreme settings: low-short and high-low; (**d–h**) for the advance optimization stage. (**d**) schema for PpIX luminescence imaging; PpIX luminescence spectrum of HFF, HT1376 and 1:1 mixture of HFF and HT1376 in (**e**) box plot (***p < 0.001); (**f**) med-med; (**g**) high-long; (**h**) high-ultra-long individually.

Lastly, with the intent to improve the HAL- induced PpIX fluorescence performance, we systemically investigated the effect of exposure times and LED illumination intensities. The aim of this experiment is to improve signal to noise ratio, between cells and background, as well as enhancing the difference in fluorescence intensity between cancer and healthy cells such as benign epithelial cells, neutrophils. In previous works, the exposure time was set at 600 ms and LED intensity was 60% as “medium-medium” (med-med). Here we explored combinations above and below these set values, total of 9 patterns, as shown in Fig. 6a. The spectra of HAL-induced PpIX fluorescence in normal fibroblast HFF and bladder cancer HT1376 are presented in Fig. 6b. These fluorescence intensities vary significantly with changes in the exposure time more so than with adjusting the LED intensity. For the purpose of diagnosis, we note that a separation between the maximum (peak) fluorescence intensity of HFF and HT1376 was seen across all illumination conditions tested. However, overlapping distributions were observed in all conditions. When we compared the extreme conditions to illustrate the difference, we can see that the separation of maxima between HFF and HT1376 was increased. In “low-short”, the separation was 200 units. While in “high-long”, the separation was 1000 units. Yet, despite an increasing separation was observed, overlapping still exists in both instances (Fig. 6c).

The preliminary results demonstrated an increased separation between non cancer HFF and bladder cancer HT1376 at high illumination intensity and long exposure time. To further evaluate the potential for photodynamic diagnosis ex vivo*,* a mixture of HFF and HT1376 cells was added to compare any fluorescence changes. In addition, the extra setting of “high-ultra-long” with 100% LED intensity and 2500 ms exposure was used to study the ultimate capacity on our system. Note that the current setting “med-med” was included as a reference in this test (Fig. 6d). The representative luminescence spectra (Fig. 6e–h) revealed that the longer exposure times increase the difference between HFF and HT1376 individually. Yet the statistical differences between the three conditions investigated here did not increase, as shown in Fig. 6e where p < 0.001 in all cases. The mixed HFF and HT1376 cells possessed a shift in peak maximum rather than two separate peaks. This indicated that the mixed cells have a big variance of fluorescence which is correlated to the different cellular uptake of HAL. Cell to cell interaction could affect the PpIX production mechanism, a hypothesis that warrant further investigations as it would significantly complicate the applicability to patient samples.

## Discussion

Blue light cystoscopy using 5-ALA as a photosensitiser prodrug has significantly higher bladder cancer detection rate than white light cystoscopy^47,48^. However, 5-ALA has low lipophilicity which limits it cellular uptake. The use of ester derivatives, such as HAL, has helped overcoming this limitation of 5-ALA. HAL-induced fluorescence cystoscopy has since increased the effectiveness of PDD in bladder cancer patients from 56 to 80% specificity^49^. Yet, blue light cystoscopy remains an invasive technique associated with risks of infection, bleeding and bladder damage.

For this reason, exogenous administration of 5-ALA and HAL in urine sediment has been tested for the non-invasive PDD of bladder cancer. In these works, the urine sediment was treated with 5-ALA or HAL at 37 °C for 2 h before measuring PpIX fluorescence using a spectrophotometer. The sensitivity of this fluorescence cytology was between 74 and 90.4% with specificity ranging from 70 to 100%^50–54^, noting that in the two studies with comparably high diagnostic performance (sensitivity: 86.9% & 90.4%, specificity: 96.2% & 100%) the slides were evaluated by experienced pathologists^53,54^. Among these studies, only one directly compared the performance of 5-ALA and HAL in the same assay, finding that HAL-induced fluorescence cytology had a higher accuracy than 5-ALA^52^. The authors attributed the false positive results obtained with 5-ALA induced fluorescence cytology to bacterial contamination, inflammation and undesirable PDD conditions, also finding that fluorescent porphyrins were not produced by bacteria when HAL was used^55^. We therefore decided to use HAL as the fluorescence inducing agent in our newly developed microfluidic chip, combining immunocapture with the PDD principle in an attempt to improve the performance of the non-invasive approach liquid biopsy approach. Optimized ex vivo processing conditions for HAL concentration and incubation time on cell monolayers were identified in our previous works^18^. Here we addressed the need to refine the protocol for optimum differences in fluorescence intensity when our platform is used with cell suspensions in condition compatible with common clinical practice. In this study, we investigated several potential obstructions to the use of our diagnostic device in clinical trials. The PpIX fluorescence of HT1376 bladder cancer cells were found to decreases in a time dependent manner over 6 h incubation in PBS whilst their cell viability remained rather constant over the same time period. Temperature gradient down to 4 °C following the addition of HAL decreased the PpIX fluorescence intensity so significantly that it became impossible to distinguish healthy cells from cancer cell lines. Together these results suggest that in order to best differentiate cancer cells from healthy cells, the specimen should be processed with the shortest possible transit times, followed by incubation with HAL in a controlled temperature environment (37 °C). In contrast, storage of the HAL premix reagent at 4 °C provided a better performance in terms of the cells subsequent PpIX fluorescence than storage at − 20 °C. The cellular PpIX fluorescence of cancer cell lines was comparable for cells bound to the different microchannel coating, namely the biocompatible polyoxazoline-based layer and the anti-EpCAM functionalized surface. These results indicate that the different cell-surface binding mechanism do not affect the production of PpIX significantly enough for it to compromise the use of POx as a positive control surface. However, multiple blue light exposure did cause PpIX photobleaching in bladder cancer cells. Therefore, we recommend to follow a protocol comprising of a single blue light exposure in order to maximise the PpIX fluorescence of cancer cells. This study also attempted to improve the discrimination between non cancer and cancer cells by optimizing imaging conditions. The separation between the peak fluorescence intensities (histogram maximum) of cancer and non-cancer cells improved with increased exposure time and illumination intensity, albeit not increasing the statistical differences between PpIX fluorescence measurement in healthy and cancer cells, and not resulting in a large enough separation for two distinct peaks to be resolved when the cells were in a mixed suspension of both healthy and cancer cells. It is worth noting that chlorin-type protoporphyrin (Ppp), but also photolabile pigments such as bilirubin and biliverdin^56^ may be formed as by-products of PpIX photobleaching. In cells, Ppp typically emits around 670 nm and its intensity is influenced by the wavelenght of the excitation light^57^. While the emission wavelength was here set at 610 nm, the filter used in the system is a long-pass filter which would also detect such higher emission events. The partial shift of the fluorescence intensity (without complete spectral separation) that is observed in the histograms could therefore be due to Ppp and other by product of PpIX photodegradation. Further optimizations of HAL processing for cell differentiation are therefore required to enhance the detection efficiency.

Furthermore, while investigations conducted on culture cell lines allowed for the protocol to be optimized step-wise in a controlled laboratory environment, future works focusing on patient urine samples are required to explore the transferability of the findings presented here.

## Conclusions

In summary, a novel non-invasive diagnostic platform that integrates the surface immunofunctionalisation techniques and photodynamic detection approach was developed. This platform can be modified to comprise a wide range of biomarkers and adapted for point-of-care use. We demonstrated the potential issues and utility of using HAL in patient sample on our platform. Further optimizations of HAL processing for cell differentiation are required to enhance the detection efficiency. While results presented in this work offer new practical directions for the handling of the urine specimen and reagents, clinical trials are needed to evaluate other potential limitations of this approach, including the presence of immune cell which may also display PpIX fluorescence as well as the possibility to detect other urogenital cancer in a specific manner or distinguish between cancer grades.

## Methods

### Materials

Hexaminolevulinate (HAL) hydrochloride and phosphate-buffered saline (PBS) tablets were purchased from Sigma-Aldrich (NSW, Australia). Nuclear red LCS1 (Cat# 17542) as a cell-permeant nucleic acid detection dye, was obtained from AAT Bioquest (CA 94085, USA). Polyclonal goat anti-human EpCAM antibody (AF960) was purchased from R&D Systems (MN 55413, USA). Skim milk powder was obtained from Merck (NSW, Australia). Polymethylmethacrylate (PMMA) microchannels slides were manufactured by Motherson Innovations (SA, Australia).

### Sensor plasma polymerization and functionalization

Polymethylmethacrylate (PMMA) slides with 3 microchannels grooves were coated via plasma deposition using a custom made plasma reactor following established procedures^17^. Briefly, the reactor was brought under vacuum, 2.e^-2^ mbar, and the substrates primed via air plasma for 3 min. The 2-methyl-2-oxazoline precursor (Sigma-Aldrich, NSW, Australia) was then inserted with a 1.2e^−1^ mbar pressure for 4 min with a continuous radiofrequency power of 20 W, 13.96 MHz^26^. The polyoxazoline (POx) coated slides were vacuumed sealed until further use^58^.

10 µg/mL of anti-EpCAM solution was added to each POx coated microchannel and stored in 4 °C overnight for functionalization. The next day, the functionalized (anti-EpCAM) slides were incubated with 1 mg/mL of skim milk solution at 37 °C for 1 h. The skim milk solution is used as a blocking agent to inhibit any non-specific cell binding. The microchannels were gently rinsed with PBS three times. Fresh PBS was added and the microchannel slide was kept at room temperature until use (within 2 h).

### Cell culture

Human fetal foreskin normal fibroblast HFFs was obtained from Cell lines service, DKFZ (Germany). Three human bladder carcinoma cell lines HT1376 (Cat# 87032402), HT1197 (Cat# 87032403) and RT4 (Cat# 91091914), were all supplied by the European Collection of Cell Cultures (ECACC; Salisbury, UK), and purchased from CellBank Australia (Westmead, NSW, Australia). DMEM from Thermo Fisher Scientific (VIC, Australia) was used to culture HFF cells. HT1376 and HT1197 cells were cultured in MEME + 1% MEM non-essential amino acid solution and RT4 cells were cultured in McCoy’s 5A, both culture media were obtained from Sigma-Aldrich (NSW, Australia). All media contained 10% fetal calf serum and 1% (v/v) penicillin/streptomycin. Cells were cultured at 37 °C in humidified 5% CO_2_ atmosphere.

### Cell viability and corresponding HAL-induced PpIX fluorescence measurement

HFF and HT1376 wells were trypsinised for 5 to 10 min. After trypsinisation, serum-containing medium was added to stop further tryptic activity. The trypsinised cell suspensions were centrifuged at 1500 rpm for 5 min. The supernatant was discarded and the cell pellet was resuspended in PBS. Cells were kept in 4 °C for different time points (1, 2, 4 and 6 h), before incubation with or without 50 µM HAL and 0.5 µM nuclear red dye for 2 h at 37 °C in the dark. After incubation, cell viabilities were determined by trypan blue assay. 100 µL of the cell suspensions were then aliquoted into 96 wells plate for PpIX fluorescence measurement on an inverted fluorescent microscope.

### HAL premix shelf-life test

A solution with concentration of 50 µM HAL and 0.5 µM nuclear red dye was prepared in 50 mL 1X PBS. The mixed reagent solution was carefully mixed at room temperature and dispensed in tubes of 1.25 mL aliquots. The aliquots were divided in two lots stored at 4 °C and − 20 °C in the dark for up to 15 months in order to investigate the reagent mixture shelf-life.

The tests consisted of a fresh mixed solution of nuclear red and HAL, a premix solution that had been kept at 4 °C and one kept at − 20 °C. At the scheduled aging time point, the premix solutions were used and tested with trypsinised cells in 1:1 ratio. Cells were incubated for 1 h at room temperature under dark condition. 500 µL of the settled cell pellet was then collected and resuspended. The stained cells were pipetted into the sensor POx coated microchannels and left for 45 min at room temperature in the dark. After 45 min, the microchannel slides were imaged (Fig. 3a).

### Cellular PpIX fluorescence in microfluidic channels and light exposure

Cells were trypsinised and incubated with freshly prepared mixed solution of HAL and nuclear red, as described above. The factors tested were (i) single vs double blue light exposure in anti-EpCAM microchannels and (ii) single blue light exposure in anti-EpCAM vs pristine POx microchannels. The stained cells were pipetted into all microchannels. For double blue light exposure (slide E1 only), a first set of images were taken immediately after injection in the microchannel, referred to as “before wash” images. Then all slides were kept in the dark for 45 min at room temperature. The microchannels were then rinsed with PBS to remove all loosely bound cells before taking “after wash” images (Fig. 5a).

### PpIX fluorescence measurement

Specifically, PpIX fluorescence was imaged through a custom-made filter cube set with excitation at 405/20 nm and emission through a long pass 610 nm emission filter. Images were analyzed by Image-Pro Premier software (Media Cybernetics Inc., MD 20852, USA). Using built-in software thresholding settings, PpIX fluorescent cells that were distinguishable from the background pixels through the long pass filter were counted. The mean intensity of each object in triplicate (n = 3) were recorded in arbitrary units defined by the software. Results were expressed as mean ± standard deviation (SD) of the mean. Data were compared by unpaired t-test or one-way ANOVA test (Minitab 18), considered statistically significant when p < 0.05. The intensities of all objects counted in each condition and each cell line were used to construct a normalized histogram, the intensity histogram corresponds to the average statistical distribution in each sample.

### Optimisation of PpIX brightness by fluorescence imaging system

Cells were trypsinised and treated with 50 µM HAL and 0.5 µM nuclear red dye for 1 h at room temperature. The stained cells were pipetted to the sensor POx coated microchannels and left for 45 min at room temperature in the dark. After 45 min, the microchannel slides were imaged. The exposure time tested ranged from 400 to 1000 ms (ms) with 30 to 100% of LED illumination intensities.

## References

[1] NICE (2017). Bladder cancer: Diagnosis and management of bladder cancer. BJU Int..

[2] Babjuk M (2019). European association of urology guidelines on non-muscle-invasive bladder cancer (TaT1 and carcinoma in situ): 2019 update. Eur. Urol..

[3] Witjes JA (2013). The impact of recurrent non-muscle-invasive bladder cancer on progression. Eur. Urol..

[4] Raitanen MP (2002). Differences between local and review urinary cytology in diagnosis of bladder cancer. An interobserver multicentre analysis. Eur. Urol..

[5] Freifeld Y, Lotan Y (2019). Effect of blue-light cystoscopy on contemporary performance of urine cytology. BJU Int..

[6] Siravegna G, Marsoni S, Siena S, Bardelli A (2017). Integrating liquid biopsies into the management of cancer. Nat. Rev. Clin. Oncol..

[7] Costa JL, Schmitt FC (2019). Liquid biopsy: A new tool in oncology. Acta Cytol..

[8] Crowley E, Di Nicolantonio F, Loupakis F, Bardelli A (2013). Liquid biopsy: Monitoring cancer-genetics in the blood. Nat. Rev. Clin. Oncol..

[9] Tian F, Liu C, Lin L, Chen Q, Sun J (2019). Microfluidic analysis of circulating tumor cells and tumor-derived extracellular vesicles. TrAC Trends Anal. Chem..

[10] Raposo G, Stoorvogel W (2013). Extracellular vesicles: Exosomes, microvesicles, and friends. J. Cell. Biol..

[11] De Rubis G, Rajeev Krishnan S, Bebawy M (2019). Liquid biopsies in cancer diagnosis, monitoring, and prognosis. Trends Pharm. Sci..

[12] Alix-Panabières C, Pantel K (2013). Circulating tumor cells: Liquid biopsy of cancer. Clin. Chem..

[13] Vaidyanathan R, Soon RH, Zhang P, Jiang K, Lim CT (2019). Cancer diagnosis: From tumor to liquid biopsy and beyond. Lab. Chip..

[14] Huang Q (2018). Nanotechnology-based strategies for early cancer diagnosis using circulating tumor cells as a liquid biopsy. Nanotheranostics.

[15] Ghosh RK, Pandey T, Dey P (2019). Liquid biopsy: A new avenue in pathology. Cytopathology.

[16] Ostrikov, K. M. R. & Vasilev, K. in *Chemeca 2016: Chemical Engineering: Regeneration, Recovery and Reinvention* (Melbourne, 2016).

[17] Macgregor-Ramiasa M (2017). A platform for selective immuno-capture of cancer cells from urine. Biosens. Bioelectron..

[18] Chan KM (2019). Biosensor device for the photo-specific detection of immuno-captured bladder cancer cells using hexaminolevulinate: An ex-vivo study. Photodiagn. Photodyn. Ther..

[19] Shirazi HS (2020). Plasma enabled devices for the selective capture and photodynamic identification of prostate cancer cells. Biointerphases.

[20] Jordan B, Meeks JJ (2019). T1 bladder cancer: Current considerations for diagnosis and management. Nat. Rev. Urol..

[21] Chou R, Dana T (2010). Screening adults for bladder cancer: A review of the evidence for the US preventive services task force. Ann. Intern. Med..

[22] Jung A (2019). Quality of life in non-muscle-invasive bladder cancer survivors: A systematic review. Cancer Nurs..

[23] Babjuk M (2017). EAU guidelines on non muscle-invasive urothelial carcinoma of the bladder: Update 2016. Eur. Urol..

[24] Satyal U, Srivastava A, Abbosh PH (2019). Urine biopsy-liquid gold for molecular detection and surveillance of bladder cancer. Front. Oncol..

[25] Ramiasa MN (2015). Plasma polymerised polyoxazoline thin films for biomedical applications. Chem. Commun..

[26] Chan KM (2020). Functional nanothin films plasma-deposited from 2-isopropenyl-2-oxazoline for biosensor applications. Biointerphases.

[27] Chan KMGJ, Li J, Vasilev K, MacGregor M (2020). Shedding light on bladder cancer diagnosis in urine. Diagnostics.

[28] McNicholas K, MacGregor MN, Gleadle JM (2019). In order for the light to shine so brightly, the darkness must be present-why do cancers fluoresce with 5-aminolaevulinic acid?. Br. J. Cancer.

[29] Steinbach P (1995). Cellular fluorescence of the endogenous photosensitizer protoporphyrin IX following exposure to 5-aminolevulinic acid. Photochem. Photobiol..

[30] Briel-Pump A (2018). Accumulation of protoporphyrin IX in medulloblastoma cell lines and sensitivity to subsequent photodynamic treatment. J. Photochem. Photobiol. B.

[31] Daneshmand S (2018). Efficacy and safety of blue light flexible cystoscopy with hexaminolevulinate in the surveillance of bladder cancer: A phase III, comparative multicentre study. J. Urol..

[32] MacGregor M (2020). Cancer cell detection device for the diagnosis of bladder cancer from urine. Biosens. Bioelectron..

[33] Zuiverloon TCM (2012). A methylation assay for the detection of non-muscle-invasive bladder cancer (NMIBC) recurrences in voided urine. BJU Int..

[34] Wood SL, Knowles MA, Thompson D, Selby PJ, Banks RE (2013). Proteomic studies of urinary biomarkers for prostate, bladder and kidney cancers. Nat. Rev. Urol..

[35] Morrissey JJ (2015). Evaluation of urine aquaporin-1 and perilipin-2 concentrations as biomarkers to screen for renal cell carcinoma: A prospective cohort study. JAMA Oncol..

[36] Mengual L (2016). Using gene expression from urine sediment to diagnose prostate cancer: Development of a new multiplex mRNA urine test and validation of current biomarkers. BMC Cancer.

[37] Ostrikov K, Michl T, MacGregor M, Vasilev K (2019). Bladder cancer cell capture: Elucidating the effect of sample storage conditions on capturing bladder cancer cells via surface immobilized EpCAM antibody. ACS Appl. Bio Mater..

[38] Milanetto MC, Imasato H, Perussi JR (2009). The importance of protoporphyrin IX efflux for ALA-PDT dosimetry. Laser Phys. Lett..

[39] Nakanishi T, Ogawa T, Yanagihara C, Tamai I (2015). Kinetic evaluation of determinant factors for cellular accumulation of protoporphyrin IX induced by external 5-aminolevulinic acid for photodynamic cancer therapy. J. Pharm. Sci..

[40] Gómez G, Pikal MJ, Rodríguez-Hornedo N (2001). Effect of initial buffer composition on pH changes during far-from-equilibrium freezing of sodium phosphate buffer solutions. Pharm. Res..

[41] Thorat AA, Suryanarayanan R (2019). Characterization of phosphate buffered saline (PBS) in frozen state and after freeze-drying. Pharm. Res..

[42] Zuiverloon TCM, de Jong FC, Costello JC, Theodorescu D (2018). Systematic review: Characteristics and Preclinical Uses Of Bladder Cancer Cell Lines. Bladder Cancer.

[43] Moan J, Streckyte G, Bagdonas S, Bech Ø, Berg K (1997). Photobleaching of protoporphyrin IX in cells incubated with 5-aminolevulinic acid. Int. J. Cancer.

[44] Sharwani A, Alharbi FA (2014). Monitoring of photobleaching in photodynamic therapy using fluorescence spectroscopy. Gulf J. Oncol..

[45] Robinson DJ (1998). Fluorescence photobleaching of ALA-induced protoporphyrin IX during photodynamic therapy of normal hairless mouse skin: The effect of light dose and irradiance and the resulting biological effect. Photochem. Photobiol..

[46] Chan KM, Gleadle J, Vasilev K, MacGregor M (2020). Probing hexaminolevulinate mediated PpIX fluorescence in cancer cell suspensions in the presence of chemical adjuvants. Int. J. Mol. Sci..

[47] Drejer D (2017). Comparison of white light, photodynamic diagnosis, and narrow-band imaging in detection of carcinoma in situ or flat dysplasia at transurethral resection of the bladder: The DaBlaCa-8 study. Urology.

[48] Inoue K (2015). Oral 5-aminolevulinic acid mediated photodynamic diagnosis using fluorescence cystoscopy for non-muscle-invasive bladder cancer: A randomized, double-blind, multicentre phase II/III study. Photodiagn. Photodyn. Ther..

[49] Rink M (2013). Hexyl aminolevulinate-guided fluorescence cystoscopy in the diagnosis and follow-up of patients with non-muscle-invasive bladder cancer: A critical review of the current literature. Eur. Urol..

[50] Miyake M (2014). Diagnostic approach for cancer cells in urine sediments by 5-aminolevulinic acid-based photodynamic detection in bladder cancer. Cancer Sci..

[51] Nakai Y (2015). Protoporphyrin IX induced by 5-aminolevulinic acid in bladder cancer cells in voided urine can be extracorporeally quantified using a spectrophotometer. Photodiagn. Photodyn. Ther..

[52] Nakai Y (2017). Spectrophotometric photodynamic detection involving extracorporeal treatment with hexaminolevulinate for bladder cancer cells in voided urine. J. Cancer Res. Clin. Oncol..

[53] Yamamichi G (2019). High diagnostic efficacy of 5-aminolevulinic acid-induced fluorescent urine cytology for urothelial carcinoma. Int. J. Clin. Oncol..

[54] Yamamichi G (2020). High performance of 5-aminolevulinic acid-induced fluorescent selective upper tract urinary cytology. Int. J. Urol..

[55] Fotinos N, Convert M, Piffaretti JC, Gurny R, Lange N (2008). Effects on gram-negative and gram-positive bacteria mediated by 5-aminolevulinic Acid and 5-aminolevulinic acid derivatives. Antimicrob. Agents Chemother..

[56] Krieg M, Whitten DG (1984). Self-sensitized photo-oxidation of protoporphyrin IX and related porphyrins in erythrocyte ghosts and microemulsions: A novel photo-oxidation pathway involving singlet oxygen. J. Photochem..

[57] Bagdonas S (2000). Phototransformations of 5-aminolevulinic acid–induced protoporphyrin IX in vitro: A spectroscopic study. Photochem. Photobiol..

[58] MacGregor M, Sinha U, Visalakshan RM, Cavallaro A, Vasilev K (2019). Preserving the reactivity of coatings plasma deposited from oxazoline precursors − An in depth study. Plasma Process. Polym..

